# Gut microbiota dysbiosis in patients with Alzheimer’s disease and correlation with multiple cognitive domains

**DOI:** 10.3389/fnagi.2024.1478557

**Published:** 2024-11-27

**Authors:** Qionglei Chen, Jiayu Shi, Gaojie Yu, Huijia Xie, Shicheng Yu, Jin Xu, Jiaming Liu, Jing Sun

**Affiliations:** ^1^Department of Geriatrics, The Second Affiliated Hospital and Yuying Children’s Hospital of Wenzhou Medical University, Wenzhou, China; ^2^Department of Preventive Medicine, School of Public Health, Wenzhou Medical University, Wenzhou, China

**Keywords:** Alzheimer’s disease, gut microbiota, short chain fatty acids, cognitive domains, microbial biomarkers

## Abstract

**Background:**

Accumulating evidence suggested that Alzheimer’s disease (AD) was associated with altered gut microbiota. However, the relationships between gut microbiota and specific cognitive domains of AD patients have yet been fully elucidated. The aim of this study was to explore microbial signatures associated with global cognition and specific cognitive domains in AD patients and to determine their predictive value as biomarkers.

**Methods:**

A total of 64 subjects (18 mild AD, 23 severe AD and 23 healthy control) were recruited in the study. 16 s rDNA sequencing was performed for the gut bacteria composition, followed by liquid chromatography electrospray ionization tandem mass spectrometry (LC/MS/MS) analysis of short-chain fatty acids (SCFAs). The global cognition, specific cognitive domains (abstraction, orientation, attention, language, etc.) and severity of cognitive impairment, were evaluated by Montreal Cognitive Assessment (MoCA) scores. We further identified characteristic bacteria and SCFAs, and receiver operating characteristic (ROC) curve was used to determine the predictive value.

**Results:**

Our results showed that the microbiota dysbiosis index was significantly higher in the severe and mild AD patients compared to the healthy control (HC). Linear discriminant analysis (LDA) showed that 12 families and 17 genera were identified as key microbiota among three groups. The abundance of *Butyricicoccus* was positively associated with abstraction, and the abundance of *Lachnospiraceae_UCG-004* was positively associated with attention, language, orientation in AD patients. Moreover, the levels of isobutyric acid and isovaleric acid were both significantly negatively correlated with abstraction, and level of propanoic acid was significantly positively associated with the attention. In addition, ROC models based on the characteristic bacteria *Lactobacillus*, *Butyricicoccus* and *Lachnospiraceae_UCG-004* could effectively distinguished between low and high orientation in AD patients (area under curve is 0.891), and *Butyricicoccus* and *Agathobacter* or the combination of SCFAs could distinguish abstraction in AD patients (area under curve is 0.797 and 0.839 respectively).

**Conclusion:**

These findings revealed the signatures gut bacteria and metabolite SCFAs of AD patients and demonstrated the correlations between theses characteristic bacteria and SCFAs and specific cognitive domains, highlighting their potential value in early detection, monitoring, and intervention strategies for AD patients.

## 1 Introduction

Alzheimer’s disease (AD) is the most common neurodegenerative disease with progressive cognitive decline (Elahi and Miller, 2017). Although many researchers have been exploring the disease’s origins and pathogenesis of this disease, no universally accepted cause has been identified, and no effective strategies for early diagnosis and treatment for this disease were currently available (Kandimalla and Reddy, 2017; Drummond and Wisniewski, 2017; Hansson, 2021). Consequently, early detection and diagnosis were crucial in slowing the progression of the disease and reducing its prevalence and morbidity (Gonçalves et al., 2024; Barron and Molofsky, 2021). In clinical practice, the diagnosis of AD frequently undergoes neuroimaging, examination of cerebrospinal fluid, and assessment of various cognitive scales (Kazmi and Hsiao, 2023; Harris, 2023). Montreal Cognitive Assessment (MoCA) was the most common tool to evaluate cognitive function in AD patients (Pinto et al., 2019), however, it was noteworthy that the differences in the education level, cultural background, examiner’s skills and experience, examination environment, and the emotional and mental state of the subjects could interfere with the accuracy of the assessment of cognitive function (Boxer and Sperling, 2023; Migliore and Coppede, 2022). Although memory impairment is the most common symptom in AD patients, some people may exhibit atypical symptoms, such as visual–spatial, language, executive and behavioral, and motor dysfunction (Graff-Radford et al., 2021), which increases the difficulty in recognizing AD in patients without prominent memory deficits (Scheltens et al., 2021). A retrospective review reported a misdiagnosis rate as high as 53% in young-onset AD, while the rate was only 4% in patients with typical symptoms (Balasa et al., 2011). Due to the heterogeneity of clinical manifestations and the complexity of disease neuropathology, there were great differences in clinical practice, resulting in complex identification of cognitive impairment in the early stage. Currently, these tests are time-consuming, cumbersome, and require a high degree of expertise from practitioners, suggesting an urgent need for an effective tool to accurately identify cognitive decline and different cognitive domains in older adults.

Accumulating evidence supported the significant alterations in the gut microbiota composition and function of AD patients (Loh et al., 2024), and abnormal gut bacteria were closely related to the development and progression of AD (Skalny et al., 2024; Zhu et al., 2022). Our previous studies revealed the increases of pro-inflammatory bacteria and the decrease of producing-short chain fatty acids (SCFAs) bacteria in APP/PS1 mice compared to the WT mice (Pan et al., 2024; Sun et al., 2019). In a clinically randomized controlled trial (RCT), the improvement of immediate memory and delayed memory functions in healthy older population were correlated with the supplementation of *Bifidobacterium longum* BB68S (Shi et al., 2022). *Bifidobacterium* was observed in the senescence-accelerated mice, as the memory deficits were improved after receiving supplementation of a probiotic preparation comprising of *B. lactis, Lactobacillus casei, B. bifidum,* and *L. acidophilus* (Yang et al., 2020). It was reported that probiotic treatment could improve memory in traumatic brain injury mice (Amaral et al., 2024). The abundance of *Lactobacillus* in mice with impaired memory was negatively correlated with the activation of NF-ĸB signaling pathway (Jang et al., 2018), and *Ruminococcaceae_UCG_004* was prominently associated with attention deficit symptom in attention-deficit/hyperactivity disorder patients (Szopinska-Tokov et al., 2020), suggesting that the changes of different bacteria might be related to the alterations of different cognitive domains. Metataxonomic analyses showed a decrease in butyrate-producing bacterial communities and a concurrent reduction in SCFA butyrate production in the 3 × Tg-AD mice (Chilton et al., 2024). Multi-strain probiotics treatment could significantly improve cognitive impairment in the SAMP8 mice, and the mechanism may be related to the regulation of SCFAs, such as valeric acid, isovaleric acid, and hexanoic acid (Xiao-Hang et al., 2024). Although gut microbiota might be involved in the occurrence and development of AD, the relationships between key bacteria and specific cognitive domains have not been explored.

In this study, we aimed to investigate the features of the gut microbiota and metabolite SCFAs in patients with AD, and the potential correlations between characteristic gut bacteria and SCFAs and specific cognitive domains. This study provided insight into the application of gut microbiota and SCFAs signatures in early detection and intervention strategies in patients with AD.

## 2 Materials and methods

### 2.1 Study population

This study was conducted in the Department of Geriatrics, the Second Affiliated Hospital of Wenzhou Medical University from January 2022 to May 2024. The AD was diagnosed according to the guidelines established by National Institute on Aging-Alzheimer’s Association workgroups (McKhann et al., 2011). Exclusion criteria: MRI evidence of stroke, hippocampal sclerosis, local space-occupying lesion, or any cognitive ability-related disease of central nervous system (CNS); The psychiatric disorders (except mild depression) or currently treated with related medication; alcohol abuse within the last 2 years; The application of epileptic medication or hypnotic; The untreated thyroid disease or deficiency of vitamin B12 or folate; The presence of hearing or visual impairments that could influence the completion of scales; The self-reported using of probiotics or antibiotics or dieting within the last 3 months; The comorbidities such as malignant tumors, acute enteritis and severe respiratory failure. A total of 41 AD patients were included in the study and stratified based on the severity of cognitive impairment into mild AD (mAD, *n* = 18, 10 ≤ MoCA <18) and severe AD (sAD, *n* = 23, MoCA <10) groups, with consideration given to their educational levels. A group of healthy control participants (HC, *n* = 23) were also recruited during the same period. The exclusion criteria mentioned above were applied to control participants as well.

### 2.2 16 s rRNA sequencing

Fresh fecal samples were collected and then promptly stored in −80°C until analysis. Microbial DNA was extracted using OMEGA-soil DNA Kit (Omega Bio-Tek, United States). The concentration and purity the extracted DNA were detected using NanoDrop2000 UV–vis spectrophotometer (Thermo Scientific, Wilmington, United States). The V3-V4 hypervariable regions of the microbial 16 s rRNA gene were amplified using primers 338F9 (ACTCCTACGGGAGGCAGCAG) and 806R (GGACTACHVGGGTWTCTAAT). Subsequently, sequencing was performed on the Illumina MiSeq System (Illumina, United States) by Majorbio Bio-Pharm Technology Co. Ltd. (Shanghai, China). Alpha diversity was assessed using the Simpson index. The difference in alpha and beta diversity among the three groups were analyzed using the Kruskal-Wallis test. Principal coordinates analysis (PCoA), based on abund-jaccard distance algorithm, was employed to analyze variation in microbiota composition, the differences among the 3 groups were evaluated using the Analysis of Similarities (ANOSIM). Linear discriminant analysis (LDA) effect size (LEfSe) based on Kruskal-Wallis test was utilized to detect differences in species abundance among groups. Subsequently, LDA was employed to estimate the impact of these different genera on intergroup differences. Genera with LDA score > 2 were diagrammed on the bar plots. The Wilcoxon rank sun test was also used to identify significantly different taxa among groups, followed by post-hoc tests for pairwise comparisons between groups using welch-uncorrected test.

### 2.3 SCFAs analysis

Fresh fecal samples (200 mg) were collected from subjects during hospitalization, outpatient or health screening center, and added in a 2 mL centrifuge tube with internal standard (L-2-chlorophenylalanine) for grinding and low-temperature ultrasonic extraction. After centrifuging for 15 min (4°C, 13,000 g), the supernatant was used for LC–MS/MS analysis on a Thermo UHPLC-Q Exactive HF-X system equipped with an ACQUITY HSS T3 column (100 mm × 2.1 mm i.d., 1.8 μm, Waters, United States) at Majorbio Bio-Pharm Technology Co. Ltd. (Shanghai, China). Followed by the pretreatment of LC/MS raw data in Progenesis QI software (Waters Corporation, Milford, United States), the metabolites were identified by searching database including the HMDB,^1^ Metlin^2^ and Majorbio Database^3^. The final concentrations of SCFAs were expressed as micrograms per milligram of feces (μg/g).

### 2.4 Statistical analysis

The differences in both the total score and individual item scores of MoCA between groups of AD patients were analyzed using Wilcoxon rank sum test. The *p* value <0.05 was considered statistically significant. The microbiota dysbiosis index was calculated by Wilcoxon rank sun test and visualized through a violin plot. Several important genera, with gradient alteration among groups or extruding in comparation of groups or correlating with scores had been selected and presented separately by bar plot. Furthermore, Spearman’s correlation analysis was performed to reveal the relationships of microbiota and SCFAs with cognitive functions. The receiver operator characteristic (ROC) curves were plotted to estimate the distinguishability of differential microbiota and SCFAs in terms of abstraction and orientation capabilities.

## 3 Results

### 3.1 Alterations of gut microbial diversity in AD patients

**Figure 1:**
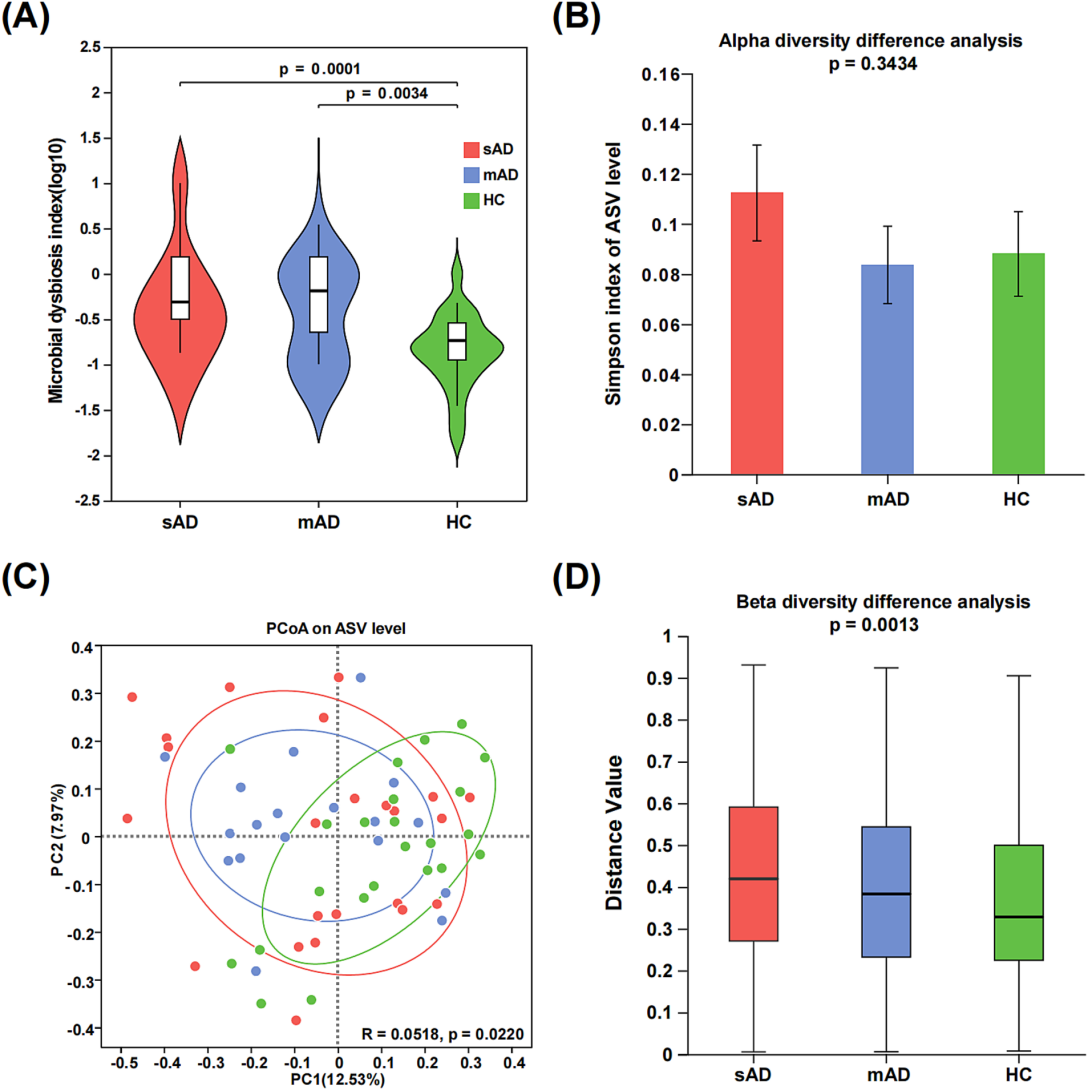
Alterations of gut microbial diversity in AD patients. (A) The MDI of the gut microbiota among sAD patients, mAD patients and HC groups. (B) The alpha diversities of gut microbiota among three groups were represented through Simpson index. (C) PCoA of gut microbiota based on abund_jaccard showed that most of the microbial samples were clustered by disease status (PC1 = 12.53%, PC2 = 7.97%). (D) The beta diversities of fecal microbiota among three groups (Green, HC; blue, mAD; red, sAD).

The microbiota dysbiosis index was significantly higher in both the sAD patients and mAD patients compared to the HC (Figure 1A, sAD vs. HC: *p* = 0.0001; mAD vs. HC: *p* = 0.0034). There was no significant difference in the Simpson index among the HC, mAD patients and sAD patients (Figure 1B). Moreover, PCoA revealed a significant difference among three groups (Figure 1C, *p* = 0.0220). Furthermore, there was significant difference in *β* diversity among three groups (Figure 1D, *p* = 0.0013).

### 3.2 Characteristic gut bacteria in AD patients

**Figure 2:**
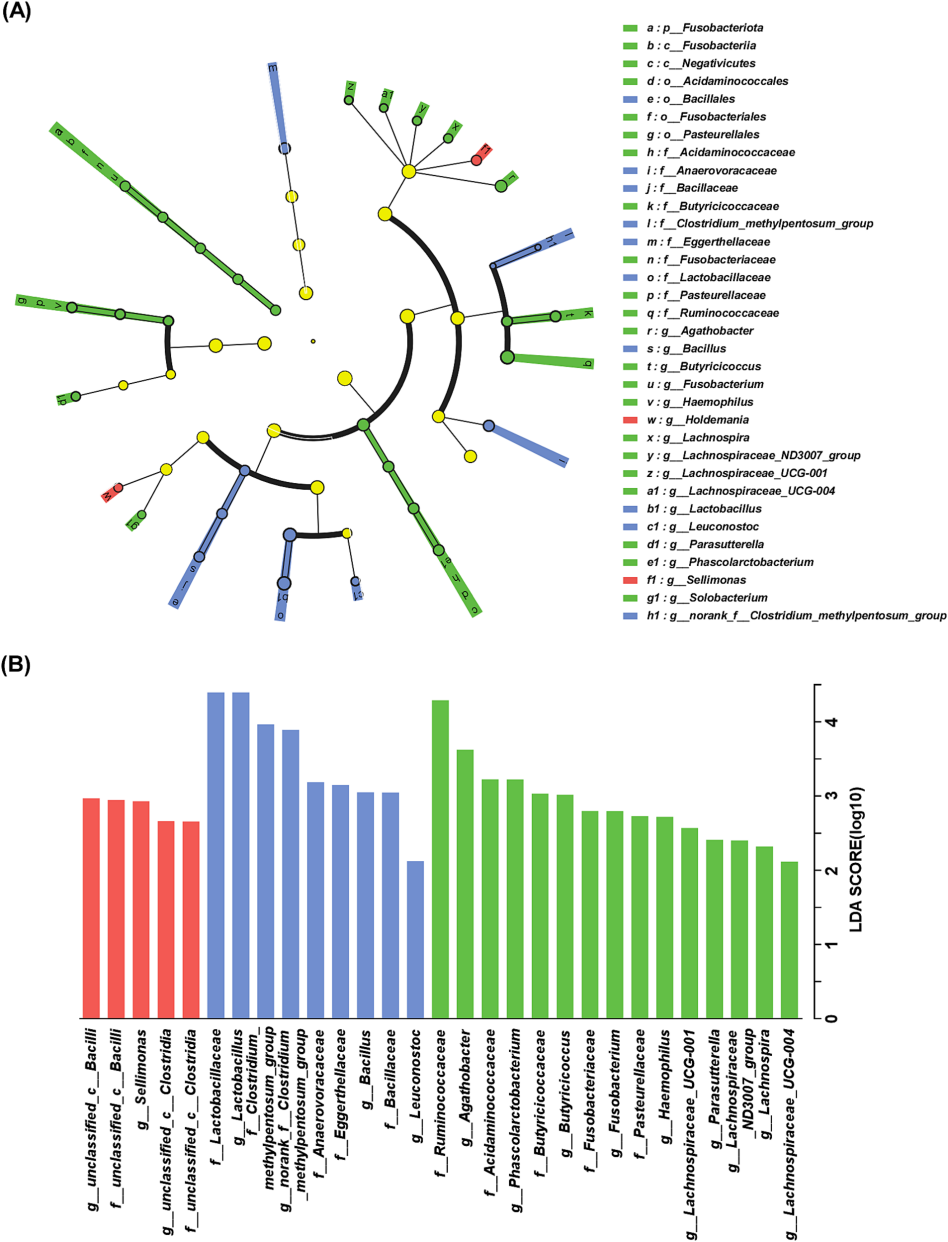
Characteristic of gut bacteria in AD patients. (A) Cladogram of distinguished bacterial populations among of mAD patients, sAD patients and HC groups from phylum to genus. (B) LDA scores of gut microbiota among sAD patients, mAD patients and HC groups. Only taxa with LDA score > 2 and *p* < 0.05 are listed. The red bar chart represented the bacteria that were more abundant in sAD patients, the blue bar chart represented a higher abundance of bacteria in mAD patients and the green bar chart represented a higher abundance of bacteria in HC groups.

The cladogram of distinguished bacterial populations was plotted in Figure 2A and there were 2 taxa, 10 taxa and 22 taxa, respectively, enriched in sAD, mAD and HC groups from phylum to genus levels. As shown in Figure 2B, LDA effect size analysis showed that 12 families and 17 genera were identified as key microbiota among three groups. Among the 29 taxa, 5 taxa were exclusively enriched in sAD patients, 9 taxa were exclusively enriched in mAD patients, and 15 taxa were exclusively enriched in HC. Significant bacteria of sAD group mainly included g_*Sellimonas*, and mAD group mainly included g_*Bacillus*, and g_*Leuconostoc*, while g_*Agathobacter*, g_*Phascolarctobacterium*, g_*Butyricicoccus*, g_*Fusobacterium*, g_*Haemophilus*, g_*Lachnospiraceae*_UCG-001, g_*Parasutterella*, g_*Lachnospiraceae*_ND3007_group, g_*Lachnospira*, and g_*Lachnospiraceae*_UCG-004 were involved in HC group.

### 3.3 Associations of gut bacteria and SCFAs with specific cognitive domains of AD patients

**Figure 3:**
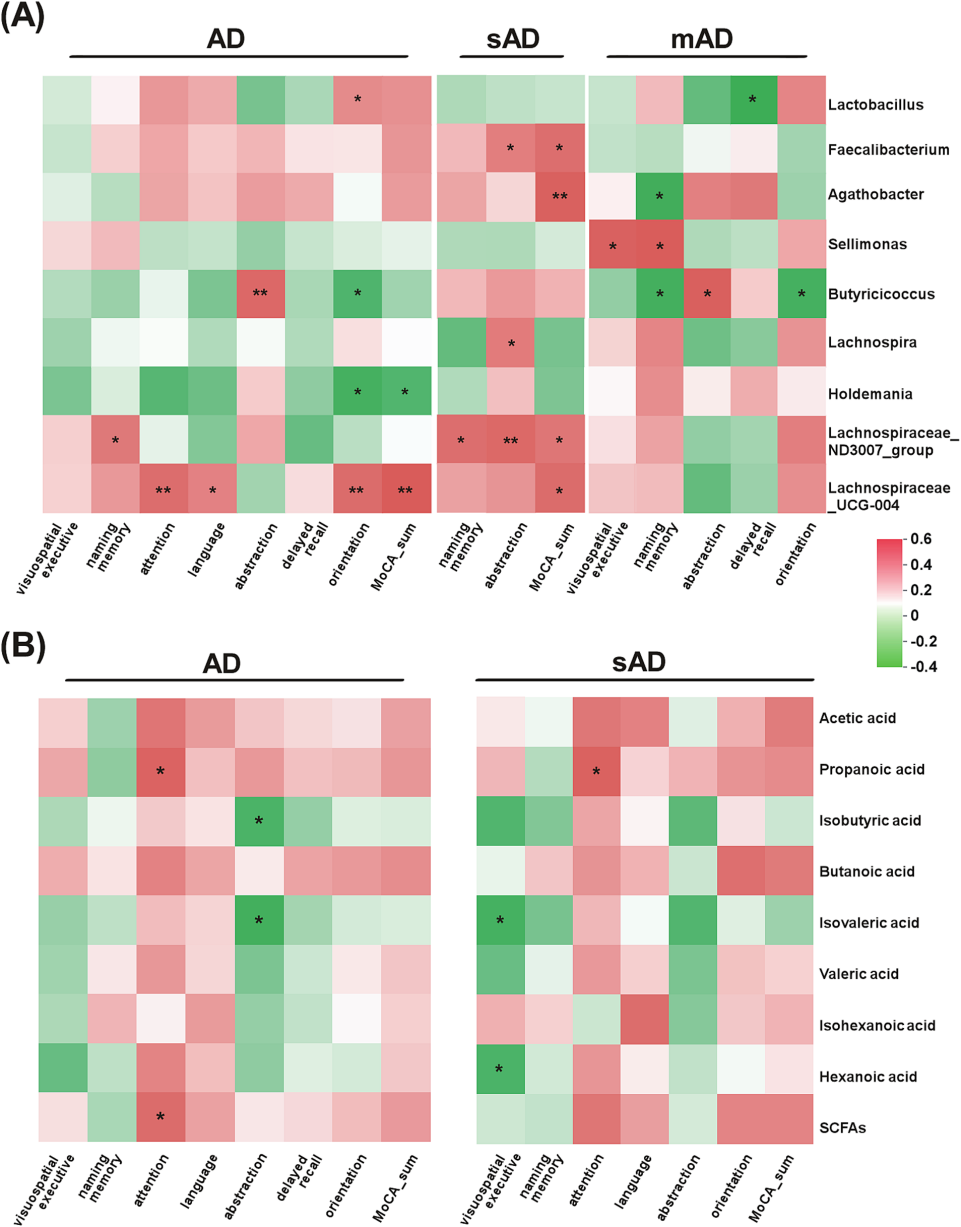
Associations of gut bacteria and SCFAs with specific cognitive domains of AD patients. (A) The heatmap of Spearman rank correlation analysis between the subitems of MoCA and gut microbiota in AD patients, sAD patients and mAD patients, respectively. (B) The heatmap of Spearman rank correlation analysis between the subitems of MoCA and metabolites in AD patients and sAD patients, respectively. Red indicated positive associations and green indicated negative associations. ^*^p < 0.05, ^**^p < 0.01.

As shown in Figure 3, the abundance of *Butyricicoccus* was positively associated with abstraction but negatively associated with orientation in mAD patients (*p* < 0.05). The abundance of *Lachnospiraceae_UCG-004* was positively associated with attention, language, orientation in AD patients (*p* < 0.05). Furthermore, the abundance of *Sellimonas* exhibited negative correlations with MoCA items in sAD patients, while showed opposite correlation in mAD patients. As for SCFAs, the levels of isobutyric acid and isovaleric acid were both significantly negatively correlated with abstraction. Additionally, the attention of AD patients was significantly positively associated with level of propanoic acid.

### 3.4 Prediction of specific cognitive domains in AD patients

**Figure 4:**
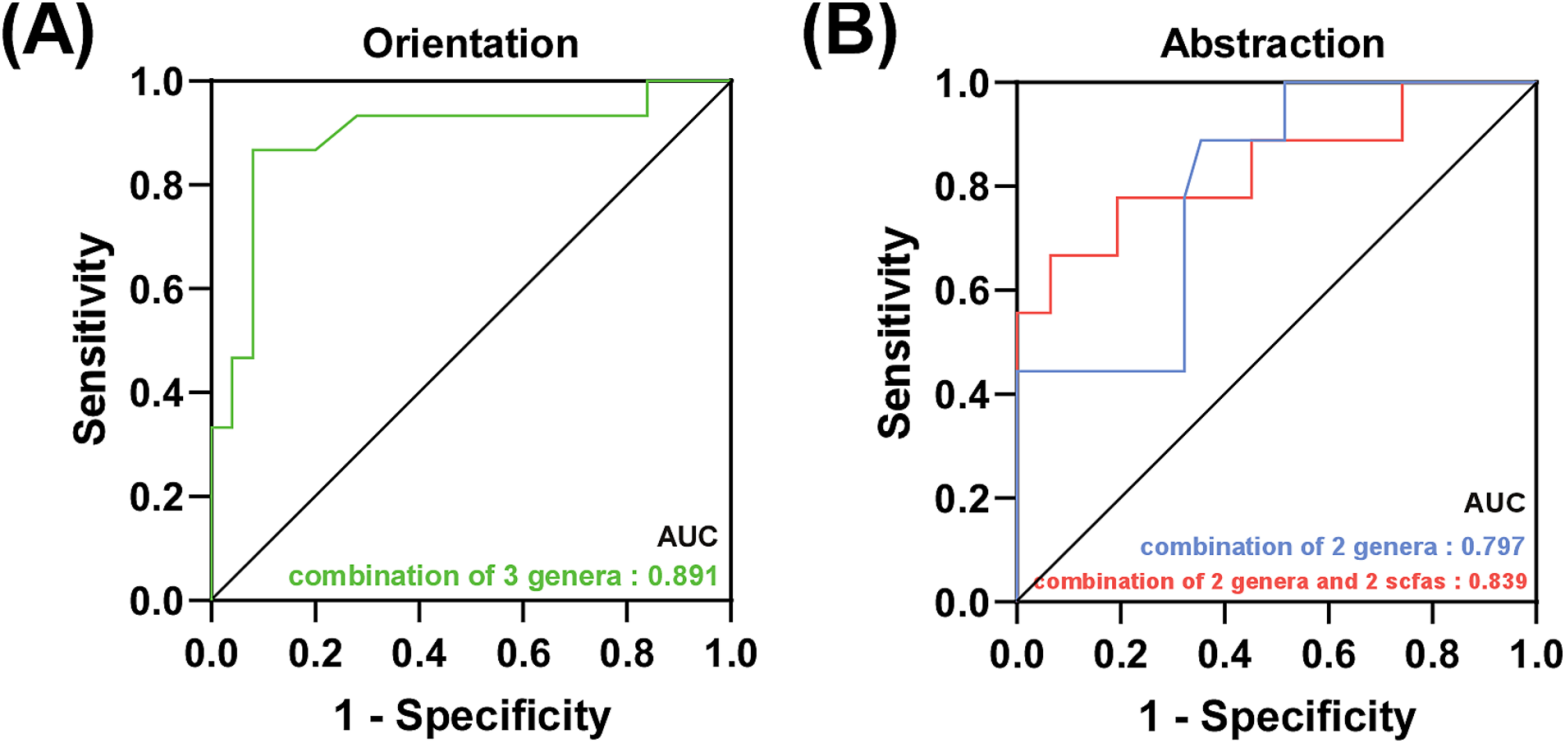
Prediction of specific cognitive domains in AD patients. (A) Green line represented the combination of 3 characteristic bacteria to distinguish the low orientation score from the high orientation score in AD patients. (B) Blue line represented the combination of 2 characteristic bacteria and red line represented the combination of 2 characteristic bacteria and 2 SCFAs to distinguish the low abstraction score from the high abstraction score in AD patients.

As shown in Figure 4, the discriminating models based on the characteristic and correlated bacteria, such as *Lactobacillus*, *Butyricicoccus* and *Lachnospiraceae_UCG-004* could effectively distinguish low orientation from high orientation (AUC = 0.891, 95% CI: 0.77–1.0, *p* < 0.01). Moreover, *Butyricicoccus* and *Agathobacter* showed a good distinction between high and low abstraction in AD patients (AUC: 0.797, 95% CI: 0.643–0.952, *p* = 0.007), when combining isobutyric acid and isovaleric acid, the AUC improved to 0.839 (95% CI: 0.667–1.0, *p* = 0.002) (See Supplementary Figures 1–3).

## 4 Discussion

In this study, we revealed the characteristics of gut microbiota and SCFAs in AD patients and demonstrated the relationships between characteristic bacteria and SCFAs and specific cognitive domains. Noteworthy, the abundance of *Butyricicoccus* was positively associated with abstraction, and the abundance of *Lachnospiraceae_UCG-004* was positively associated with attention, language, orientation in AD patients. Moreover, the levels of isobutyric acid and isovaleric acid were both significantly negatively correlated with abstraction, and level of propanoic acid was significantly positively associated with the attention. Furthermore, ROC models based on the characteristic bacteria and SCFAs exhibited good sensitivity and specificity in predicting specific cognitive domains. These results suggested that gut microbiota and metabolite SCFAs could be used as convenient biomarkers to efficiently identify the global cognition and specific cognitive domains in AD patients.

An increasing number of studies have shown that the structure and composition of gut microbiota in subjects with cognitive impairment have undergone significant changes (Zhuang et al., 2018). A previous study revealed that the feces collected from patients with mild cognitive impairment (MCI) exhibited similar variation in alpha and beta diversities (Li et al., 2019). An animal study investigating antibiotics-induced gut dysbiosis built bridges between significantly decreased Chao1 index, depleted SCFAs relative abundance and impaired object recognition memory (Fu et al., 2024), implying casual relationships between disruption of gut microbiota and specific cognitive function. Additionally, mouse models with diabetes-induced cognitive impairment showed a significant increase in alpha diversity, and improved spatial learning and memory capabilities following intermittent fasting (Liu et al., 2020). These findings suggest a link between gut dysbiosis and cognitive impairment, warranting further exploration into the precise nature of their interaction and the underlying mechanisms involved.

In this study, the abundance of *Butyricicoccus* and *Lachnospiraceae_UCG-004* could effectively distinguish between low and high orientation in AD patients, and *Butyricicoccus* and *Agathobacter* showed a good distinction between high and low orientation in AD patients. As cognitive impairment worsened, the abundance of *Lachnospiraceae*_UCG-004 gradually decreased (Yamashiro et al., 2024), consistent with our results. As for *Lachnospiraceae_UCG-004*, its changes in abundance were correlated with MoCA scores in this study. While previous studies had not directly linked *Lachnospiraceae_UCG-004* with cognitive function, its decreased abundance in PD (Nishiwaki et al., 2020), varying levels in different subtypes of schizophrenia patients (Kowalski et al., 2023), and increased richness after supplementation of prebiotics (Rodriguez et al., 2020) suggested the contributing role of *Lachnospiraceae_UCG_004* in maintaining microecological balance and overall health. In this study, *Lachnospiraceae_UCG_004* showed significantly positive correlations with the sum of MoCA in both AD patients and sAD patients with consistent trends in mAD group, indicating the potential of *Lachnospiraceae_UCG-004* as a biomarker for cognitive impairment. In this study, *Agathobacter* and *Butyricicoccus* had discriminative abilities in abstraction capability. Although there was a lack of direct research on the role of *Butyricicoccus* in abstraction capability, our heatmap analysis had indicated a significant effect of *Butyricicoccus* on cognitive abilities, particularly abstraction and orientation capabilities in mAD group.

Recent studies have emphasized the importance of SCFAs in regulating brain diseases, including AD (Yan et al., 2024; Ya-Xi et al., 2024). SCFAs are microbial metabolites, which are mainly composed of acetic acid, propionic acid and butyric acid, formate, valerate and caproate, and its production can be regulated by the gut microbiota (LeBlanc et al., 2017). A growing body of evidence suggested that altered gut microbiota could lead to changes in SCFAs levels, which were closely related to the pathogenesis and clinical manifestations of AD. The changes in SCFAs production were directly associated with alterations in the gut microbiota (Cipe et al., 2015; Zhu et al., 2011). In this study, the levels of isobutyric acid and isovaleric acid were significantly negatively correlated with abstraction, and the level of propanoic acid was significantly positively associated with attention of AD patients. In addition, the diagnostic model of AD was established using characteristic bacteria or the combination of SCFAs could distinguish abstraction in AD patients, and the AUC area had satisfactory prediction effect. As for isovaleric acid, a previous study had revealed the impaired Na^+^/K^+^-ATPase activity in cerebral cortex in the pathogenesis of isovaleric acidemia (Ribeiro et al., 2007) and the level of isovaleric acid was re-reduced after supplementation of bioactive peptide extracted from walnut protein with amended cognitive function in dementia mice (Wang et al., 2019). It was reported that the levels of propionate and isobutyric acid were decreased in AD mice (Zheng et al., 2019). A growing number of studies have demonstrated a link between increased propionate and brain disorders. Propionate had adverse effects on autism (Frye et al., 2017), depression (Szczesniak et al., 2016) and social behavior in a rodent model of autism spectrum disorder (Shams et al., 2019). SCFAs, generally considered beneficial to human physiology and exert beneficial effects through various mechanisms, linked the brain function via the microbiota-gut-brain axis. Considering that SCFAs are only related to specific cognitive domains, such as abstraction, orientation and attention, further study to explore the detailed causative relationship between SCFAs and cognitive domains is needed.

Several limitations of this study should be noted. Firstly, the sample size was small, potentially impacting the reliability of the conclusions drawn. Secondly, due to practical constraints, it was challenging to categorize patients based on individual cognitive domains. Thirdly, our study solely focused on assessing the concentration of SCFAs in fecal samples, without incorporating blood samples. Despite these limitations, our study still identified several genera correlated with specific cognitive function and showed the potential diagnostic and therapeutic ability of gut microbiota.

To conclude, these findings revealed the signatures gut bacteria and metabolite SCFAs of AD patients, and demonstrated the correlations between theses characteristic bacteria and SCFAs and specific cognitive domains, highlighting that gut microbiota and SCFAs could be used as potential biomarkers to efficiently identify the global cognition and specific cognitive domains in AD patients.

## Data Availability

The datasets presented in this study can be found in online repositories. The names of the repository/repositories and accession number(s) can be found at: https://www.ncbi.nlm.nih.gov/, PRJNA1174882.

## Footnotes

^1^ http://www.hmdb.ca/

^2^ https://metlin.scripps.edu/

^3^ https://www.majorbio.com
