# Supplementary figures and images for: Gut microbiota dysbiosis in patients with Alzheimer’s disease and correlation with multiple cognitive domains
Source: Front Aging Neurosci. 2024 Nov 27;16:1478557. doi: 10.3389/fnagi.2024.1478557 (PMC11632125; doi:10.3389/fnagi.2024.1478557)

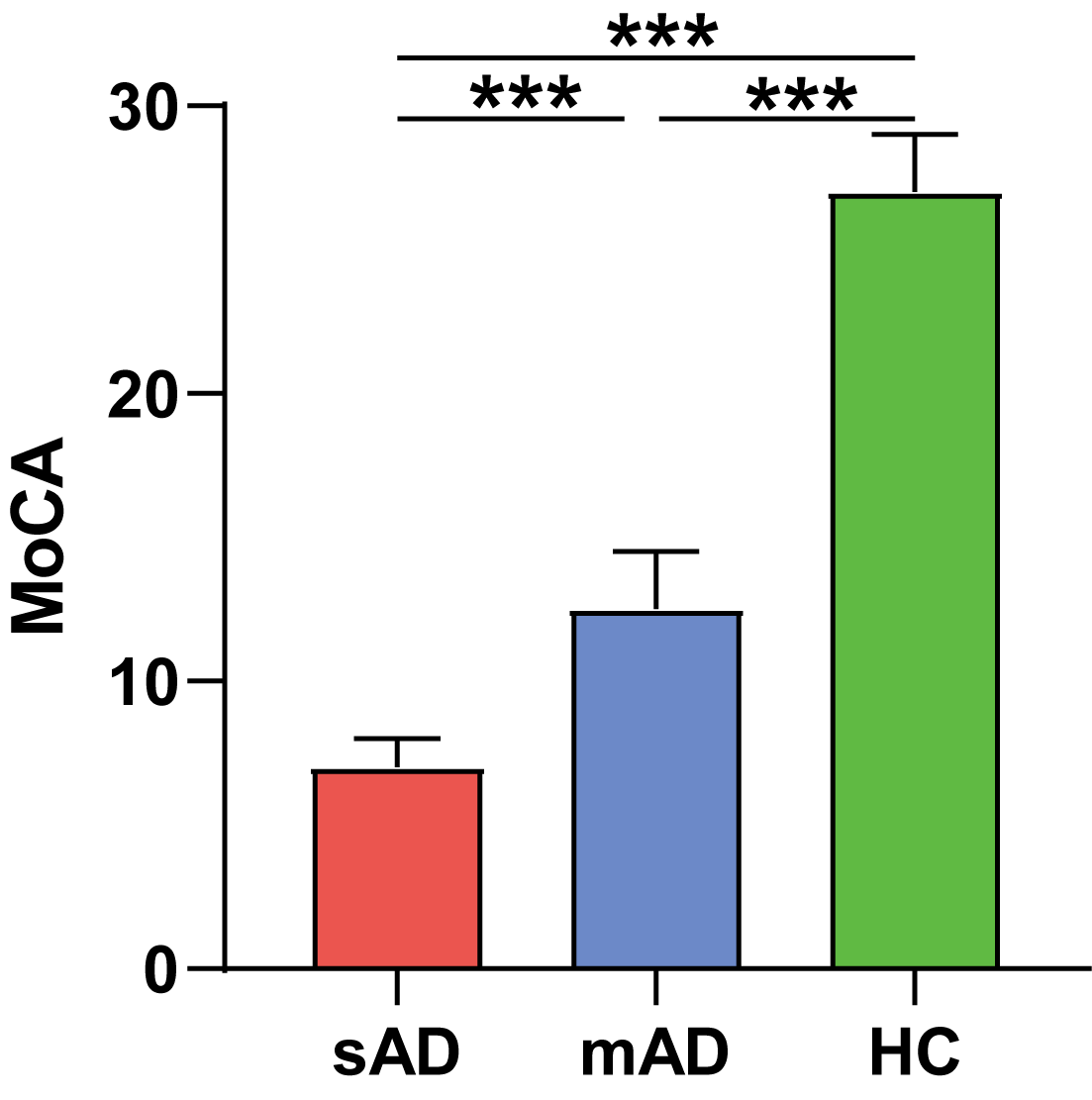

Supplement: Supplementary file 1 [file Image_1.tif]

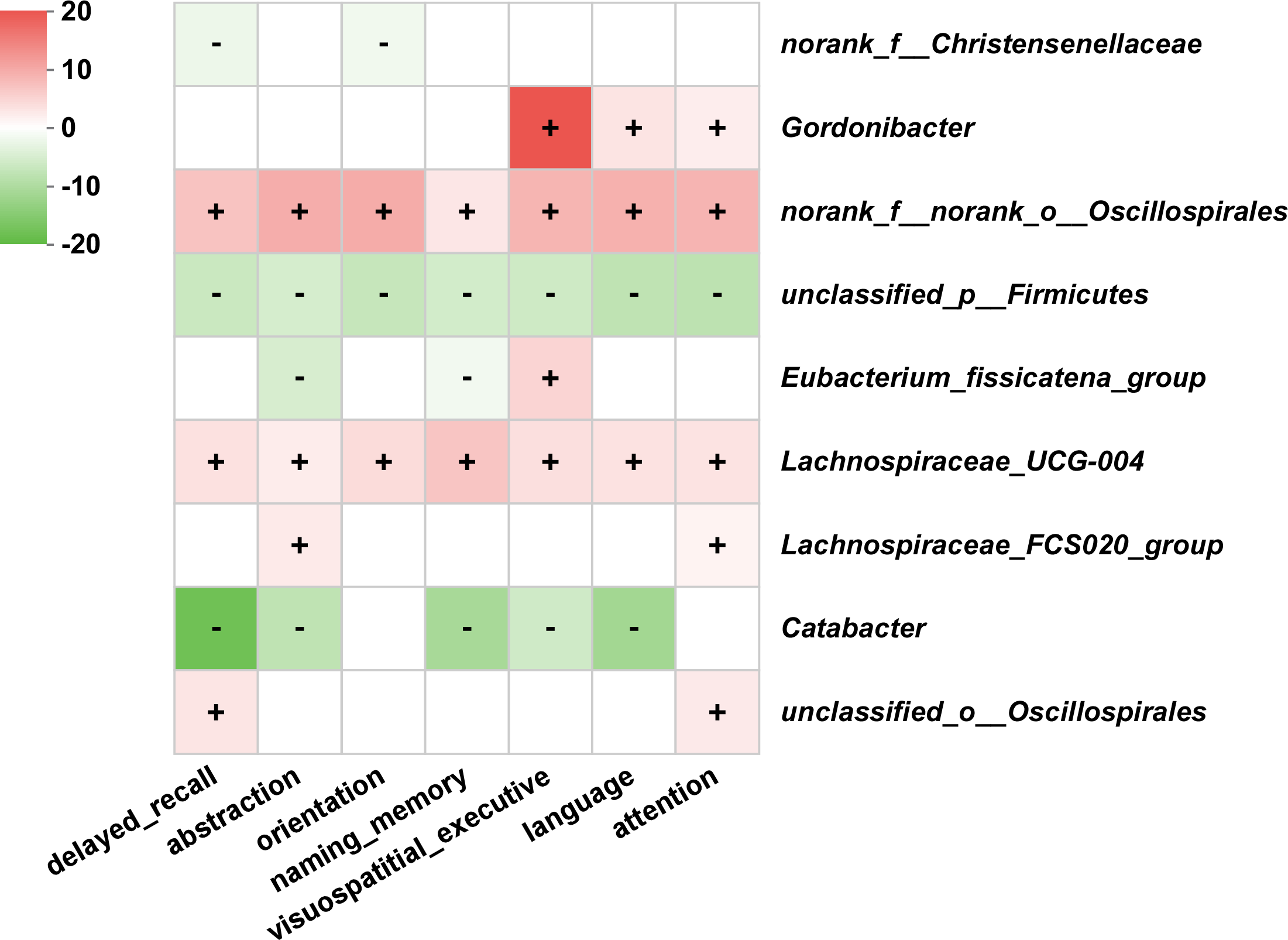

Supplement: Supplementary file 2 [file Image_2.tif]

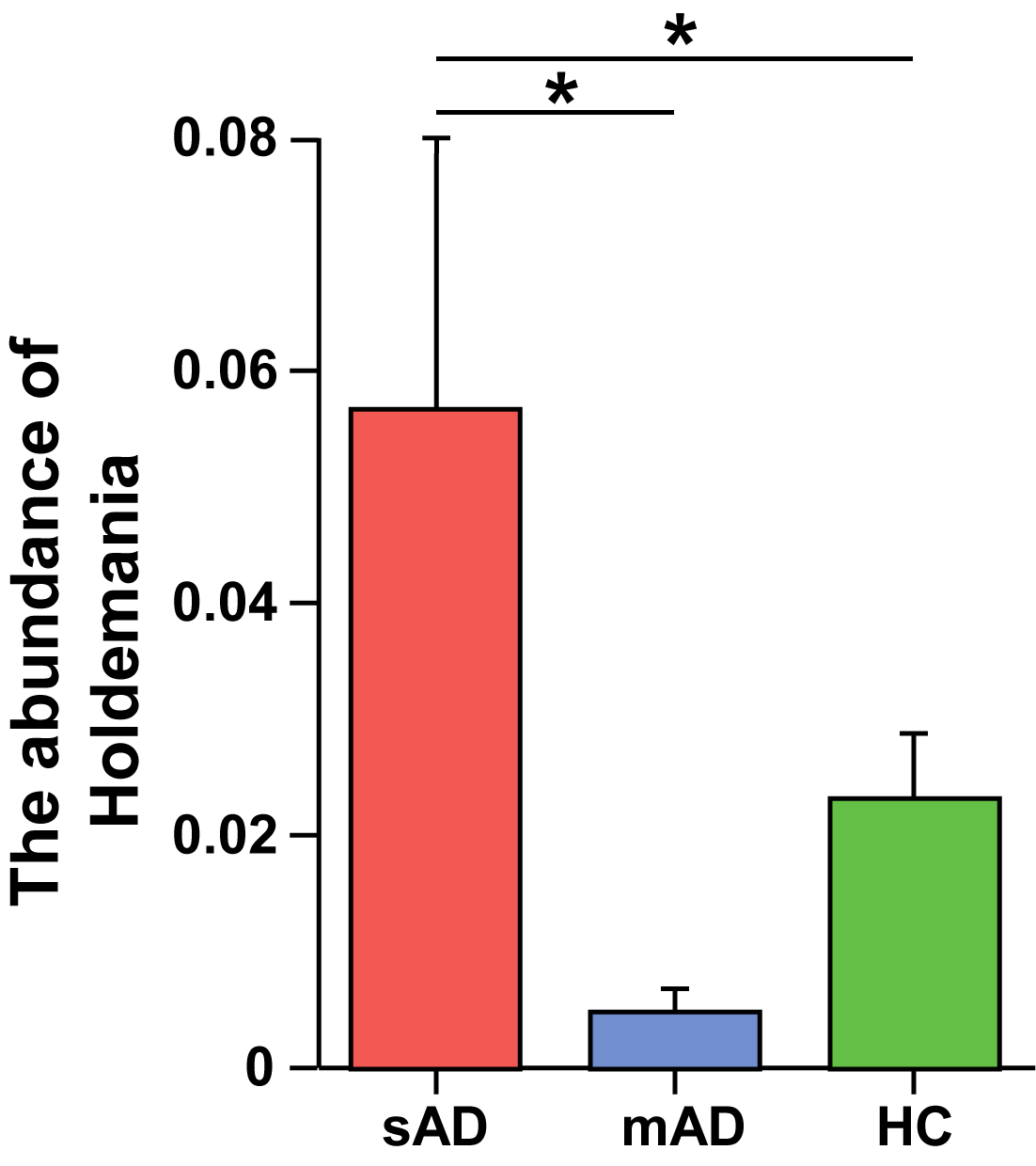

Supplement: Supplementary file 3 [file Image_3.tif]
